# Whole genome sequence data of *Paenibacillus tyrfis* YSS-72.2.G2, a chitinolytic bacterium newly isolated from a National Park of Vietnam

**DOI:** 10.1016/j.dib.2024.110087

**Published:** 2024-01-23

**Authors:** Dinh Minh Tran, Tu Oanh Do, Quang Vinh Nguyen

**Affiliations:** Institute of Biotechnology and Environment, Tay Nguyen University, Buon Ma Thuot, Dak Lak 630000, Vietnam

**Keywords:** Draft genome sequence, *Paenibacillus tyrfis*, Chitinolytic system, Antimicrobial metabolites

## Abstract

*Paenibacillus tyrfis* YSS-72.2.G2 is a soil chitinolytic bacterium newly isolated from Yok Don National Park of Vietnam. Our previous results demonstrated that this bacterium was a strong chitinase producer, possessed plant growth promotion, and had high activity against phytopathogenic fungi. However, the genome sequence of this strain is unknown. This work aimed to establish data on the genome sequence of *P. tyrfis* YSS-72.2.G2 and its chitinase system for further assessments regarding biocontrol mechanisms and plant growth promotion. The *P. tyrfis* YSS-72.2.G2 genome is 7,756,121 bp in size and 53.4 % G+C. It harbors 6,948 protein-coding genes, 5 rRNA genes, 82 tRNA genes, 4 ncRNA genes, 99 pseudo genes, and 5 CRISPR arrays. Genes involved in heavy metal resistance (5 genes), iron acquisition (5 genes), and IAA biosynthesis (5 genes) were predicted in the genome. There were 234 carbohydrate-active enzymes found in this genome; among them, 13 enzymes possibly possess activity against phytopathogens. Chitin-degrading system of YSS-72.2.G2 contains 15 chitinolytic enzymes. In addition, 28 gene clusters coding for antimicrobial metabolites were identified, of these, 14 show no sequence similarities to the known clusters. The raw sequences were submitted to the Sequence Read Archive on the National Center for Biotechnology Information with accession number PRJNA946889. The genome sequence of *P. tyrfis* YSS-72.2.G2 has been deposited in the DDBJ/GenBank/EMBL database under accession number NZ_BSDJ00000000. Data provide insight into the genomic information of strain YSS-72.2.G2. This is the first work reporting data on the genome sequence of *P. tyrfis* isolated from Vietnam.

Specifications Table

**Table:** 

Subject	Microbiology: Applied Microbiology
Specific subject area	Molecular biology, Bioinformatics
Data format	Raw, Filtered, and Analyzed
Type of data	Figures, Tables
Data collection	- Extraction of the genomic DNA of strain YSS-72.2.G2 - Preparation of Illumina library - Sequencing of the library using Illumina (2×150 PE) - Bioinformatic analysis of data
Data source location	• Institution: Institute of Biotechnology and Environment, Tay Nguyen University • City/Province/Region: Buon Ma Thuot/Dak Lak/The Central Highlands • Country: Vietnam
Data accessibility	1. Raw sequences Repository name: SRA NCBI Data identification number: PRJNA946889 Direct URL to data: https://www.ncbi.nlm.nih.gov/bioproject/PRJNA946889 2. Genome sequence Repository name: DDBJ/GenBank/EMBL Data identification number: NZ_BSDJ00000000 Direct URL to data: https://www.ncbi.nlm.nih.gov/nuccore/NZ_BSDJ00000000.1

## 1 Value of the Data

• Data elucidates the biocontrol ability and plant-growth promotion of P. tyrfis YSS-72.2.G2 isolated from the Central Highlands of Vietnam.
• Data provides an insight into the genomic information of chitinolytic bacterium P. tyrfis YSS-72.2.G2.
• Data can be useful for comparing the genome sequence of P. tyrfis YSS-72.2.G2 isolated from the Central Highlands and others.
• Data can be valuable for further examinations concerning crop production using gene expression approaches.

## 2 Background

The chitinolytic bacterium, *P. tyrfis* YSS-72.2.G2, was newly isolated from the soil sample collected from Yok Don National Park in the Central Highlands region of Vietnam. Experimental data showed that this bacterium exhibited high activities of chitinase, protease, cellulase, and amylase; produced indole acetic acid (IAA) and siderophore; and displayed high antagonistic activity (65.67 % inhibition) against phytopathogenic fungi [1]. To the best of our knowledge, no genome sequences of *P. tyrfis* have been reported from Vietnam to date. In addition, experimental studies about the chitinase system, extracellular enzymes, and secondary metabolites of *P. tyrfis* have been undocumented. This work aims to establish data on the genome sequence of *P. tyrfis* YSS-72.2.G2 and its chitinase system to provide genomic information for further evaluations regarding biofertilizer and biocontrol mechanisms.

## 3 Data Description

**Fig. 1:**
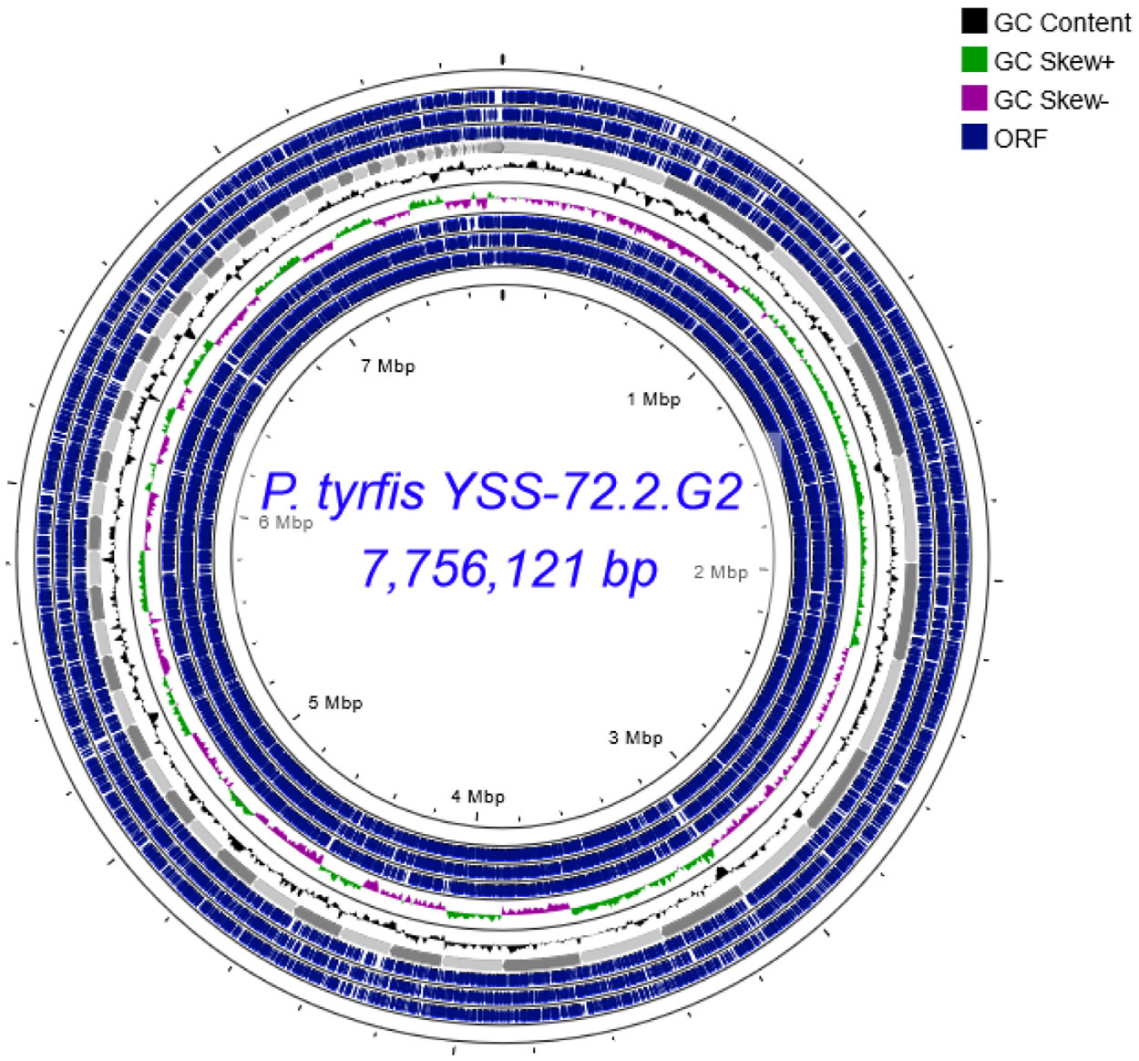
Circular representation of the *Paenibacillus tyrfis* YSS-72.2.G2 draft chromosome. The draft chromosome was generated using the Proksee server (https://proksee.ca/).

The genome of strain YSS-72.2.G2 is 7,756,121 bp in length and has a 53.4 % G+C content generated from 130 contigs (Fig. 1). Of these contigs, the maximum size was 505,185 bp, and the minimum was 207 bp. DFAST analysis showed that 6,948 protein-coding genes, 5 rRNA genes, 82 tRNA genes, 4 ncRNA genes, 99 pseudo genes, and 5 CRISPR arrays were predicted from the genome. Among the predicted proteins, there are 2,922 deduced hypothetical proteins and 4,018 functional proteins (Table 1). The raw sequences were submitted to the Sequence Read Archive on the National Center for Biotechnology Information with accession number PRJNA946889 and can be accessed at https://www.ncbi.nlm.nih.gov/bioproject/PRJNA946889. The genome sequence of P. tyrfis YSS-72.2.G2 has been deposited in the DDBJ/GenBank/EMBL database under accession number NZ_BSDJ00000000 and can be accessed at https://www.ncbi.nlm.nih.gov/nuccore/NZ_BSDJ00000000.1.

**Table 1:** Characteristics of the draft genomes of *Paenibacillus tyrfis* YSS-72.2.G2.

Parameter	*Paenibacillus tyrfis* YSS-72.2.G2
Genome size (bp)	7,756,121
G+C content (%)	53.4
No. of contigs	130
The largest contig (bp)	505,185
The shortest contig (bp)	207
N50	236,798
L50	12
Protein-coding sequences	6,948
Hypothetical proteins	2,930
Functional proteins	4,018
rRNA	5
tRNA	82
ncRNA	4
Pseudo genes	99
CRISPR arrays	5

Genome analysis showed that five genes involved in heavy metal resistance were predicted from the genome of strain YSS-72.2.G2. Among those, three genes coding for cobalt-zinc-cadmium resistance (loci YDYSG_12510, YDYSG_25980, and YDYSG_64910) and two genes involved in arsenic resistance (loci YDYSG_58000 and YDYSG_68960). In addition, five genes involved in siderophore biosynthesis (loci YDYSG_40580, YDYSG_51690, YDYSG_51700, YDYSG_51710, and YDYSG_51720) and 5 genes regulating IAA biosynthesis, including two encoding for tryptophan synthases (loci YDYSG_32640 and YDYSG_32650), two for anthranilate phosphoribosyltransferases (loci YDYSG_26220 and YDYSG_32680), and one for phosphoribosylanthranilate isomerase (locus YDYSG_32660), were identified from the *P. tyrfis* YSS-72.2.G2 genome.

Carbohydrate-active enzymes (CAZymes) are critical enzymes for using and degrading carbohydrates. Table 2 shows that 234 CAZymes were predicted from the genome of strain YSS-72.2.G2, including 108 glycoside hydrolases (GH), 58 glycosyltransferases (GT), 9 polysaccharide lyases (PL), 44 carbohydrate esterases (CE), 7 auxiliary activities (AA), and 8 carbohydrate-binding modules (CBM). Among them, 13 enzymes (9 GH18, one GH19, 2 GH16, and one GH46) may possess activities against hyphal growth of phytopathogenic fungi and egg-hatching of plant parasitic nematodes. On the other hand, a gene encoding for serine protease (locus YDYSG_38780), 2 genes for cysteine proteases (loci YDYSG_35860 and YDYSG_58880), 4 genes for metalloproteases (loci YDYSG_31490, YDYSG_36300, YDYSG_40660, and YDYSG_55430), 2 genes for cellulases (loci YDYSG_02610 and YDYSG_06360), and 4 genes for glucoamylases (loci YDYSG_13190, YDYSG_22240, YDYSG_43570, and YDYSG_62150) were identified in the genome of strain YSS-72.2.G2.

**Table 2:** Carbohydrate-active enzymes found in the *Paenibacillus tyrfis* YSS-72.2.G2 genome.

Class	Family (No.)
Glycoside hydrolases	GH2 (3), GH3 (3), GH4 (4), GH5 (2), GH8 (2), GH9 (1), GH10 (1), GH13 (6), GH15 (4), GH16 (2), GH18 (9), GH19 (1), GH20 (3), GH23 (2), GH26 (3), GH29 (4), GH30 (1), GH31 (2), GH32 (1), GH35 (1), GH38 (2), GH39 (1), GH46 (1), GH57 (1), GH63 (1), GH65 (1), GH68 (1), GH73 (1), GH85 (2), GH87 (1), GH88 (3), GH94 (1), GH95 (3), GH105 (2), GH109 (22), GH117 (1), GH123 (1), GH125 (1), GH126 (1), GH129 (1), GH136 (2), GH151 (1), GH154 (1), GH171 (1)
Glycosyltransferase	GT1 (6), GT2 (26), GT4 (14), GT5 (1), GT17 (1), GT26 (1), GT28 (4), GT35 (1), GT51 (4)
Polysaccharide lyases	PL6 (1), PL8 (1), PL9 (1), PL15 (1), PL17 (2), PL31 (3)
Carbohydrate esterases	CE1 (9), CE4 (23), CE7 (2), CE9 (4), CE14 (5), CE17 (1)
Auxiliary activities	AA1 (1), AA3 (1), AA4 (1), AA6 (1), AA7 (1), AA10 (2)
Carbohydrate-binding modules	CBM9 (1), CBM13 (1), CBM32 (3), CBM35 (1), CBM50 (1), CBM54 (1)

**Fig. 2:**
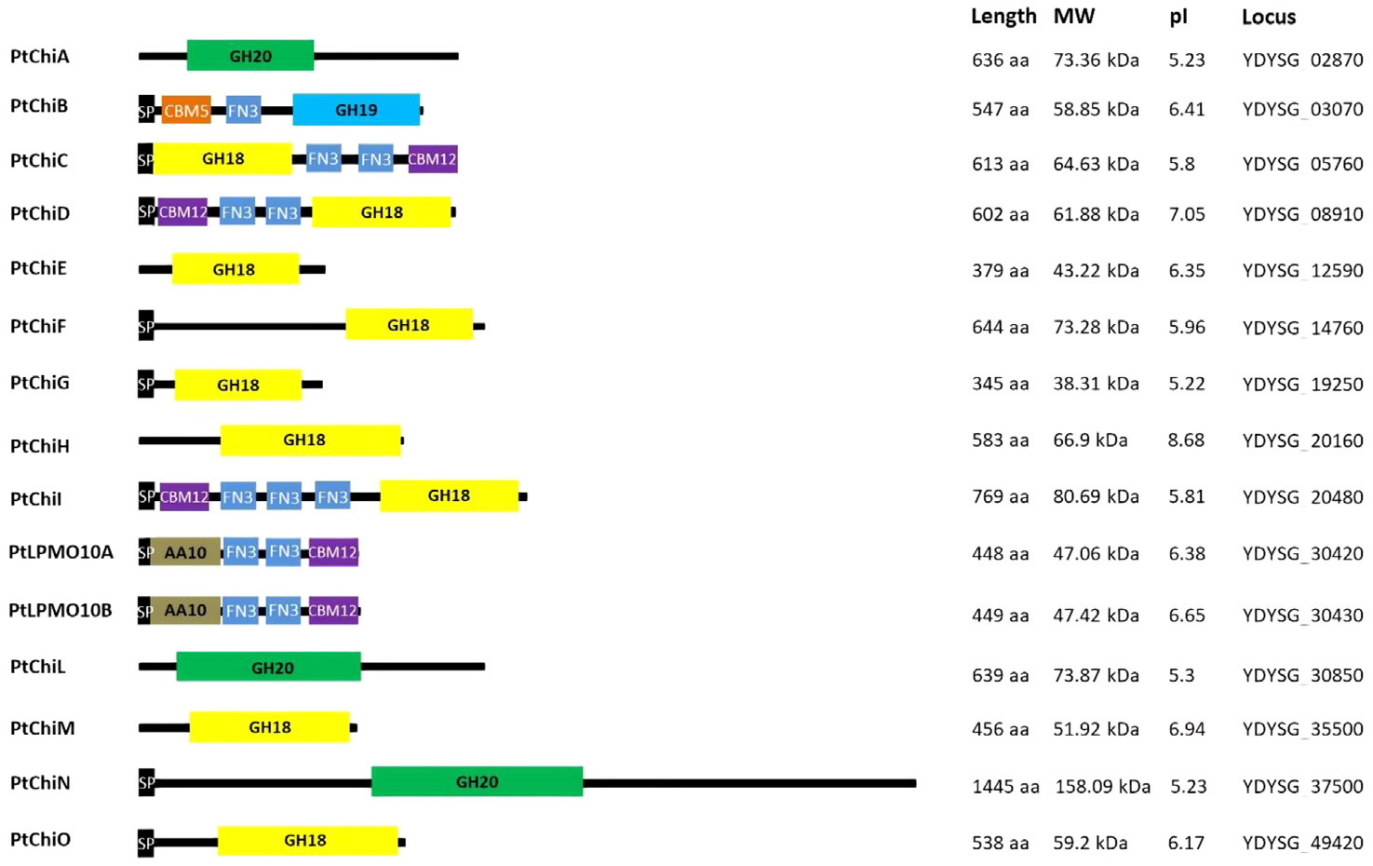
Chitinolytic system of *Paenibacillus tyrfis* YSS-72.2.G2. SP, signal peptide sequence; GH20, the catalytic domain belonging to GH20 chitinases; GH19, the catalytic domain belonging to GH19 chitinases; GH18, the catalytic domain belonging to GH18 chitinases; AA10, the catalytic domain belonging to AA10 proteins. MW, molecular weight; pI, isoelectric point; aa, amino acid; kDa, kilodaltons. PtChiA to PtChiO, chitinase A to chitinase O of *P. tyrfis* YSS-72.2.G2.

Fig. 2 shows that strain YSS-72.2.G2 possesses 15 genes encoding chitinolytic enzymes. Among these genes, 9 encode GH18 chitinases, one encodes GH19 chitinase, 3 encode GH20 β-*N*-acetylglucosaminidases, and 2 encode auxiliary activity family 10 proteins (AA10) of lytic polysaccharide monooxygenases. Of the chitinolytic enzymes, six contain functional domains, including carbohydrate-binding module family 5 (CBM5), fibronectin type III domain (FN3), and carbohydrate-binding module family 12 (CBM12), and 10 have the signal peptide for secretion of the enzymes outside *P. tyrfis* YSS-72.2.G2 cells.

**Fig. 3:**
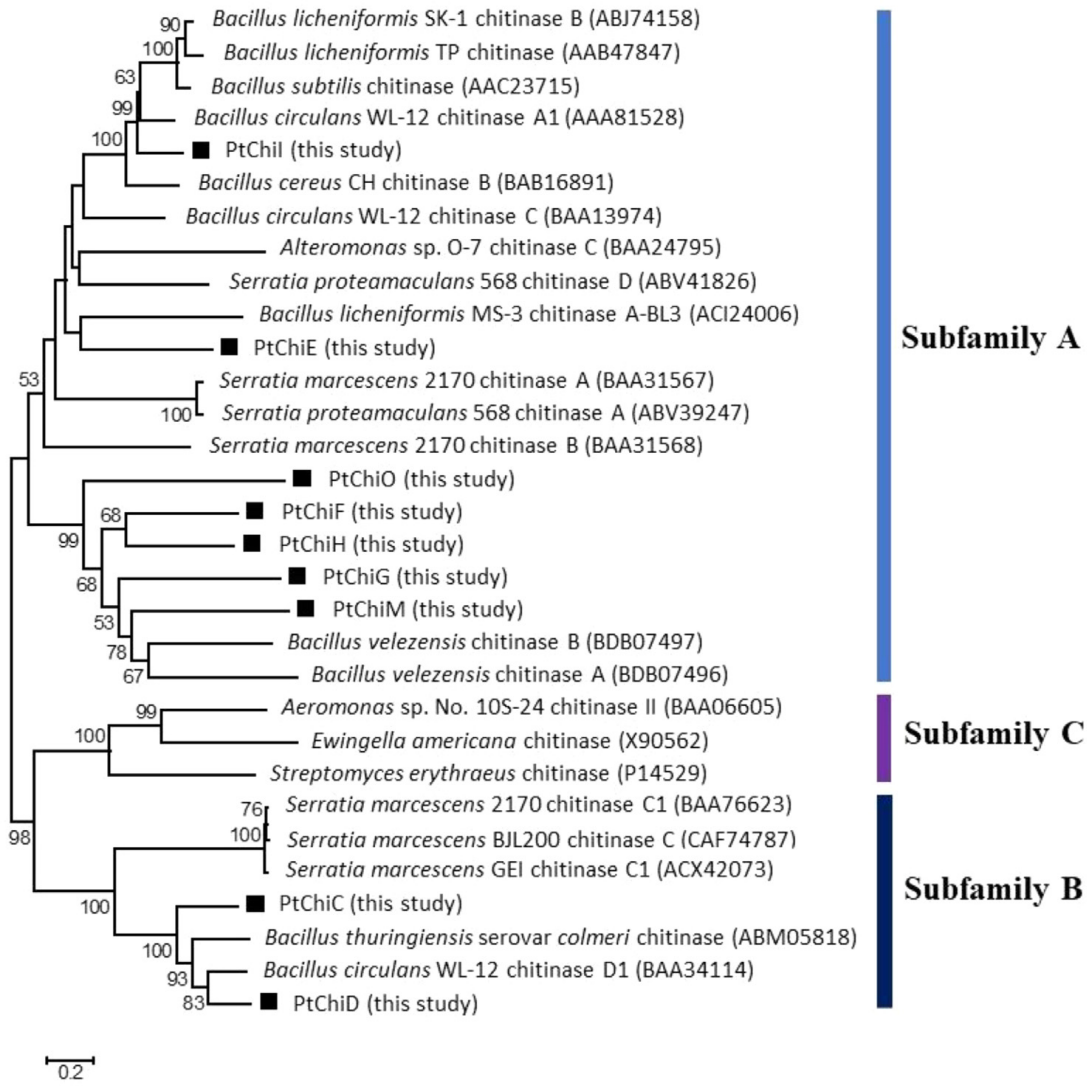
Phylogenetic analysis of bacterial family 18 chitinases. Bar, 0.2 substitutions per amino acid position. PtChiC to PtChiO, chitinase C to chitinase O of *P. tyrfis* YSS-72.2.G2.

Bacterial GH18 chitinases are grouped into three subfamilies (A, B, and C). These subfamilies play different properties in the hydrolysis of chitin. Subfamily A chitinases usually show high chitinase activity against crystalline chitin compared to subfamilies B and C [2]. As shown in Fig. 3, seven chitinases of strain YSS-72.2.G2 were grouped into subfamily A, and two chitinases (PtChiC and PtChiD) were clarified into subfamily B.

**Fig. 4:**
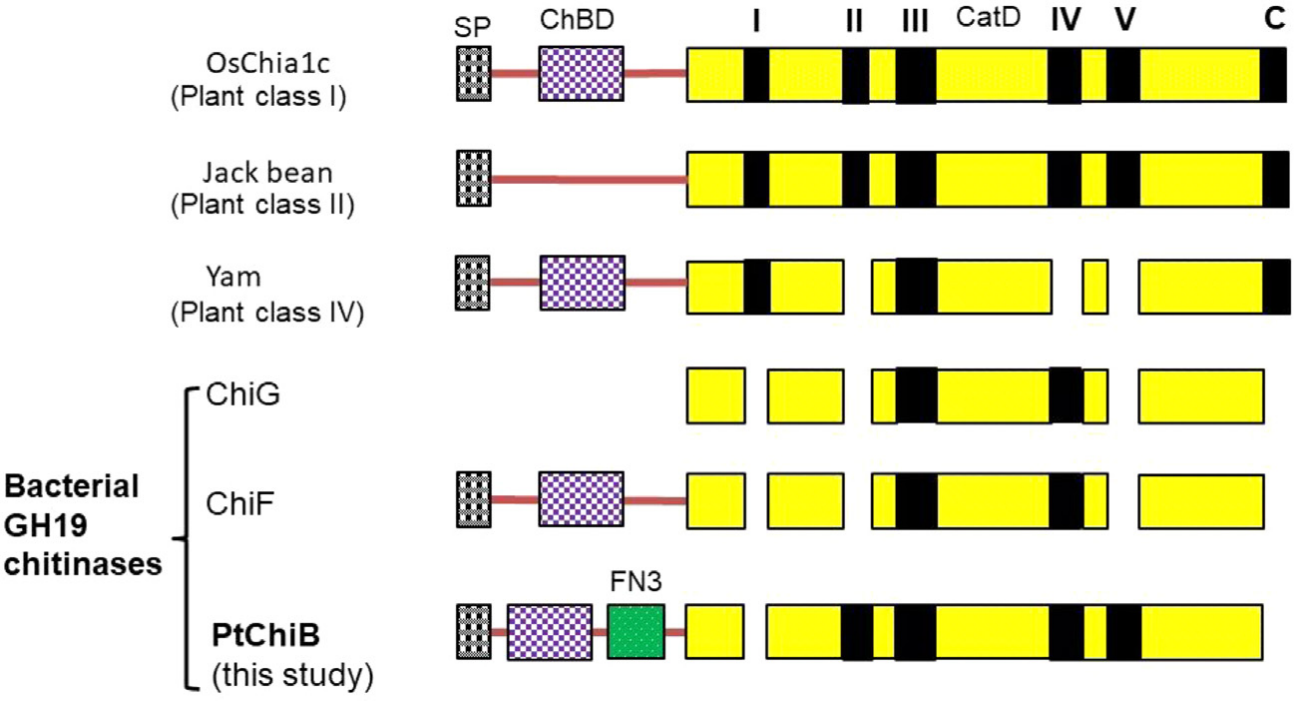
Schematic representation of the loop structure of the GH19 chitinases. I–V and C with black squares, loops I–V and C in catalytic domains. SP, signal peptide; ChBD, chitin-binding domain; CatD, catalytic domain; FN3, fibronectin type III domain. OsChia1c, class I chitinase (BAA03751) of rice; Jack bean, class II chitinase (CAA07413) of jack bean; Yam, class IV chitinase (BAC56863) of yam; ChiG, chitinase G of Streptomyces *coelicolor* A3(2) (BAA75648); ChiF, chitinase F of *S. coelicolor* A3(2) (BAA75646); PtChiB (this study), chitinase B of *P. tyrfis* YSS-72.2.G2. *Note:* The figure presents the loop structure of the family 19 chitinase, only, and does not consider the size of these enzymes.

It was demonstrated that plant family 19 chitinases are subdivided into three classes (I, II, and IV) based on the arrangement of the loop structure and organization of the domain. Differences in the loop structure of chitinases can affect the substrate binding activity of each chitinase, and therefore, chitinases may show a difference in enzymatic properties and biological function. Bacterial GH19 chitinases have no loops I, II, V, and the C-terminal loop [3], [4]. As shown in Fig. 4, the loop structure of PtChiB of strain YSS-72.2.G2 was different from that of reported plant and bacterial GH19 chitinases. The catalytic domain of PtChiB has loops II, III, IV, and V compared to characterized bacterial GH19 chitinases, which contain loops III and IV. Compared to GH19 chitinases of plant classes I and II, which have loops I, II, III, IV, V, and the C-terminal, the catalytic domain of PtChiB lacks only loop I.

**Fig. 5:**
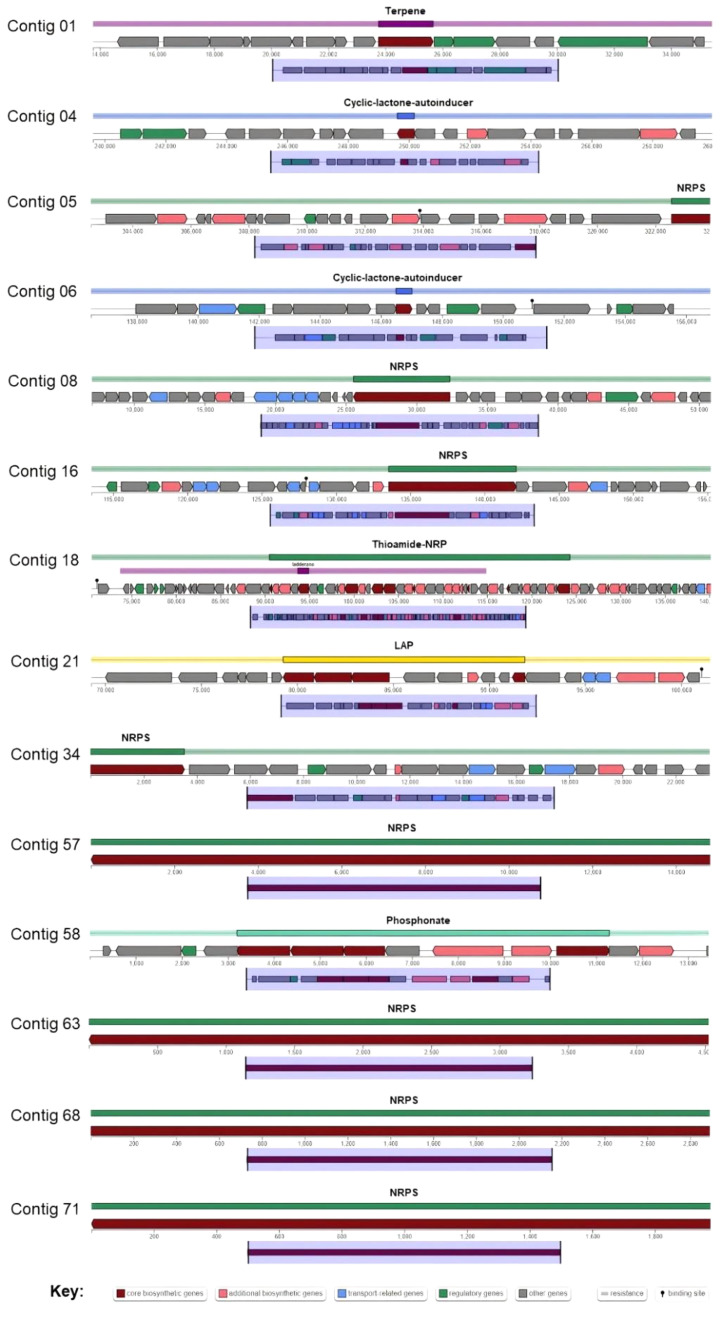
Organization of putative novel biosynthetic gene clusters found in the *Paenibacillus tyrfis* YSS-72.2.G2 genome.

The ability to produce antimicrobial metabolites is important for the biocontrol of plant pathogens. Antimicrobial metabolites are clarified into three classes: polyketides, nonribosomal peptides, and ribosomally synthesized post-translationally modified peptides [5]. This work found 28 gene clusters responsible for antimicrobial metabolite biosynthesis from the *P. tyrfis* YSS-72.2.G2 genome (Table 2). Among them, 14 show no identities to the known clusters (Table 3 and Fig. 5).

**Table 3:** Biosynthetic gene clusters of *Paenibacillus tyrfis* YSS-72.2.G2.

Contig	Location (nucleotide to nucleotide)	Metabolite type	Most similar known cluster	Similarity (%)
Contig 01	13,759−35,669	Terpene	–	–
Contig 01	260,722−282,792	Redox-cofactor	Lankacidin C	13
Contig 01	355,993−414,020	NRPS/T1PKS	cystothiazole A	11
Contig 02	2−202,513	NRPS/T1PKS/TransAT-PKS	Brevicidine	18
Contig 02	288,208−391,338	NRPS/TransAT-PKS	Paenilipoheptin	7
Contig 04	239,621−260,178	Cyclic-lactone-autoinducer	–	–
Contig 05	302,560−324,118	NRPS	–	–
Contig 06	136,496−157,017	Cyclic-lactone-autoinducer	–	–
Contig 08	7,016−51,311	NRPS	–	–
Contig 08	238,447−284,384	NRPS/Lanthipeptide-class-i	Penisin	100
Contig 10	165,117−187,264	Opine-like-metallophore	Bacillopaline	100
Contig 16	113,542−155,675	NRPS	-	-
Contig 17	1−35,743	NRPS	Tridecaptin	80
Contig 18	70,525−140,929	Ladderane/Thioamide-NRP	–	–
Contig 21	69,274−101,861	LAP	–	–
Contig 24	2,210−65,951	NRPS	Fusaricidin B	87
Contig 27	1,932−100,512	NRP-metallophore/NRPS	Bacillibactin	100
Contig 34	1−23,531	NRPS	–	–
Contig 36	1−27,176	NRPS	Octapeptin C4	41
Contig 49	17,485−42,657	NRPS	Octapeptin C4	64
Contig 57	1−14,978	NRPS	–	–
Contig 58	1−13,587	Phosphonate	–	–
Contig 60	1−10,537	NRPS	Paenilarvin A	75
Contig 63	1−4,574	NRPS	–	–
Contig 68	1−2,921	NRPS	–	–
Contig 69	1−2,147	NRPS	Thermoactinoamide A	100
Contig 70	1−2,052	NRPS	Thermoactinoamide A	100
Contig 71	1−1,998	NRPS	–	–

*Note:* -, sequence similarity not found.

## 4 Experimental Design, Materials and Methods

### 4.1 Genomic DNA extraction, library preparation, and genomic sequencing

Bacterial cells of *P. tyrfis* YSS-72.2.G2 were cultured on Luria–Bertani agar plate at 30 °C for 24 h. The extraction of the genomic DNA was performed using the QIAamp DNA mini kit (Qiagen, Germany), according to the instructions of the manufacturer. High-quality DNA (OD_260/280_ = 1.8 to 2.0) was used for the next steps. Genomic libraries were prepared using the Nextera XT DNA Library Preparation Kit (Illumina, USA) per the manufacturer's instructions. Finally, prepared libraries were used for sequencing (2×150 Paired-End) using V3 chemistry (Illumina, USA) with the Illumina MiSeq platform [1].

### 4.2 Genomic assembly, functional annotation, and data analysis

Guppy 3.0.3 was used for base calling in order to get quick sequencing reads. Trimmomatic 0.36 [6] was used to trim the reads. Unicycler 0.4.8 [7] was used to assemble the edited sequences, and Pilon 1.23 [8] was used to polish them. Ultimately, the web-based annotation pipeline known as DFAST [9] was used to automatically annotate the draft genome sequence. The draft genome was submitted to the DNA Data Bank of Japan (DDBJ) database. CAZymes were analyzed using the dbCAN2 metaserver [10]. Phylogeny of deduced chitinases was analyzed using MEGA 6.0 software [11]. Putative gene clusters responsible for antimicrobial metabolite biosynthesis were predicted using antiSMASH 6.0 [12].

## Limitations

Not applicable.

## Ethics Statement

The current work does not involve human subjects, animal experiments, or any data collected from social media platforms.

## CRediT authorship contribution statement

**Dinh Minh Tran:** Conceptualization, Methodology, Investigation, Formal analysis, Software, Data curation, Validation, Visualization, Writing – original draft, Writing – review & editing. **Tu Oanh Do:** Investigation, Formal analysis, Software. **Quang Vinh Nguyen:** Data curation, Validation, Visualization.

## Data Availability

Paenibacillus tyrfis strain YSS-72.2.G2, whole genome shotgun sequencing project (Original data) (NCBI). Genome sequence of Paenibacillus tyrfis YSS-72.2.G2 reveals a complete chitinolytic system and novel secondary metabolites (Original data) (NCBI).
